# Neurobrucellosis in a common bottlenose dolphin (*Tursiops truncatus*) stranded in the Canary Islands

**DOI:** 10.1186/s12917-019-2089-0

**Published:** 2019-10-21

**Authors:** Eva Sierra, Antonio Fernández, Idaira Felipe-Jiménez, Daniele Zucca, Gabriella Di Francesco, Josué Díaz-Delgado, Simona Sacchini, Miguel A. Rivero, Manuel Arbelo

**Affiliations:** 1 Division of Histology and Animal Pathology, Institute for Animal Health and Food Security (IUSA), Veterinary School, Universidad de Las Palmas de Gran Canaria, 35416 Arucas, Gran Canaria, Canary Islands Spain; 2 National and international Reference Laboratory for Brucellosis, Istituto Zooprofilattico Sperimentale Abruzzo e Molise, Teramo, Italy; 3 Laboratory of Wildlife Comparative Pathology, School of Veterinary Medicine and Animal Science, University of São Paulo, São Paulo, SP Brazil; 4 Texas A&M Veterinary Medical Diagnostic Laboratory (TVMDL), College Station, TX USA

**Keywords:** *Brucella*, Bottlenose dolphin, Canary Islands, *Cetacean Morbillivirus*, Neurobrucellosis

## Abstract

**Background:**

*Brucella* spp. isolation is increasingly reported in cetaceans, although associated pathologies, including lesions of the musculoskeletal and nervous systems, are less frequently described. Concerning the nervous system, *Brucella* sp. infection causing meningitis, meningoencephalitis or meningoencephalomyelitis have been extensively reported in striped dolphins (*Stenella coeruleoalba*), and less frequently in other cetacean species.

**Case presentation:**

A juvenile female common bottlenose dolphin (*Tursiops truncatus*) was found stranded alive in Lanzarote (Canary Islands, Spain) in 2005, but died shortly after. On physical examination, the dolphin showed a moderate body condition and was classified as code 2 (fresh dead) at the time of necropsy. The main gross findings were severe multiorgan parasitism, thickened and congested leptomeninges, and (sero)fibrino-suppurative and proliferative arthritis of the shoulder joint. Histopathological examination revealed the distinct features of a sub-acute systemic disease associated with *Cetacean Morbillivirus* (CeMV) infection. However, brain lesions diverged from those reported in systemic CeMV infection. This led to suspect that there was a coinfecting pathogen, based on the characteristics of the inflammatory response and the lesion distribution pattern in the central nervous system. *Brucella* sp. was detected in the brain tissue by PCR and *Brucella* antigen was demonstrated by immunohistochemistry in the brain and shoulder joint lesions.

**Conclusions:**

The zoonotic potential of marine mammal strains of *Brucella* has been demonstrated both in natural and laboratory conditions. In this study, PCR detected *Brucella* sp. in the brain of a common bottlenose dolphin stranded in the Canary Islands; the dolphin was also co-infected with CeMV. This is the first detection of *Brucella* sp. infection in a stranded cetacean in this archipelago. Therefore, we stress the importance of taking adequate measures during the handling of these species to prevent the transmissions of the infection to humans.

## Background

Brucellosis is a zoonotic disease widely described in terrestrial mammals and in an increasing number of marine counterparts [1, 2]. There have been many reported cases of *Brucella* spp. infection in marine mammals worldwide since its first simultaneous description (in cetaceans and pinnipeds) in 1994 [3, 4]. Based on their host specificity, two species of *Brucella* have been described in marine mammals: *B. ceti*, with cetaceans as its preferred hosts, and *B. pinnipedialis*, which mainly infects pinnipeds [5]. Antibodies against *Brucella* spp. have been detected in a wide range of cetacean species (at least 53) [1, 2, 6–9], although *Brucella*-associated pathological findings have been reported less frequently. Specifically, the pathological findings described in common bottlenose dolphins (*Tursiops truncatus*) infected with *Brucella* spp. included: blubber abscesses [10], discospondylitis and vertebral osteomyelitis [11, 12], placentitis, and abortion [4, 13], and parasitic pneumonia and lung abscesses [14–16]. *Brucella* spp. infection associated with meningitis, meningoencephalitis or meningoencephalomyelitis has been widely reported in striped dolphins (*Stenella coeruleoalba*) [12, 17–24], and less frequently in other cetacean species: Atlantic white-sided dolphin (*Lagenorynchus acutus*) [25], short-beaked common dolphin (*Delphinus delphis*) [26], harbour porpoise (*Phocoena phocoena*) [27], long-finned pilot whale (*Globicephala melas*) [28], sperm whale (*Physeter macrocephalus*) [29], and common bottlenose dolphin [30, 31]. In addition, *B. ceti* has also been isolated from the brain of some species with no evidence of associated pathology [18, 32], which could be due to the virulence differences exhibited by some strains of *B. ceti* [33] and/or differences in species or individual susceptibilities. In this paper, we present a confirmed report of a central nervous system (CNS) pathology associated with *Brucella* sp*.* in a common bottlenose dolphin, previously reported to be infected with *Cetacean Morbillivirus* (CeMV) [34]. This is the first case of brucellosis in a cetacean species in the Canary Islands.

## Case presentation

**Fig. 1:**
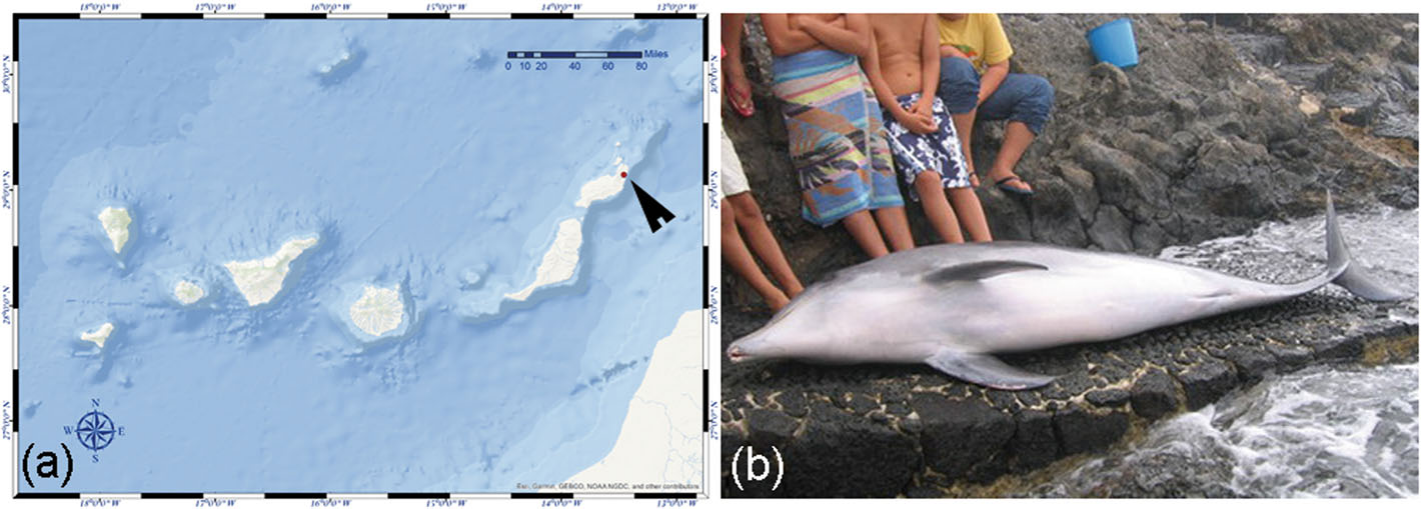
**a** Map of the stranding location (red point, arrowhead) (QGIS). **b**. Ventro-lateral view of the stranded common bottlenose dolphin

A juvenile female common bottlenose dolphin (laboratory identification number: I-225/05) was found stranded alive in Arrieta, Lanzarote (Canary Islands, Spain) in 2005, but died shortly after (Fig. 1). The total body length was 250 cm, and a moderate body condition was estimated according to morphometric parameters [35]. A complete standardised necropsy was conducted within 6 h post-mortem. Samples were collected and stored by duplicate, fixed in 10% neutral buffered formalin for histology and immunohistochemistry (IHC) analyses and frozen at − 80 °C for molecular analysis. After fixation, tissue samples were trimmed and routinely processed. The tissues were then embedded in paraffin-wax, sectioned (5 μm-thick) and stained with hematoxylin and eosin (HE) for examination by light microscopy. Immunohistochemical analysis was performed on selected formalin-fixed, paraffin-embedded (FFPE) samples of brain, intestinal, lymphatic, pancreatic, pulmonary, renal, and splenic tissues, using a monoclonal antibody against *Canine Distemper virus* (CDV), as previously described [36]. Immunohistochemical detection of *Brucella* antigen was performed on brain and shoulder joint samples using a non-commercial monoclonal antibody produced at the Institute Zooprofilattico Sperimentale dell’Abruzzo e del Molise Giuseppe Caporale. The antibody reacts with *B. melitensis* biotype 1, 2 and 3; *B. melitensis* Rev. 1; *B. abortus* biotype 2, 3 and 6; *B. ceti*; and *B. pinnipedialis*. Appropriate positive and negative IHC controls were included. The immunoreactivity observed in the positive control – a PCR-confirmed *Brucella* meningoencephalitis in a striped dolphin – consisted in macrophage-like cells harbouring bacterial antigen [Di Francesco G, Petrini A, D'Angelo AR, Di Renzo L, Luciani M, Di Febo T, et al: Immunohistochemical investigations on neurobrucellosis-affected striped dolphins (*Stenella coeruleoalba*), unpublished]. Molecular detection of CeMV was performed by a 1-step reverse transcription conventional PCR of a 426-bp conserved region of the phosphoprotein (P) gene [37]. A quantitative duplex-PCR amplifying a 150-bp fragment of the IS711 gene to detect *Brucella* at genus level and identify genotype ST27, was used for the *Brucella* PCR assay [38], which also included the shoulder joint (FFPE) sample. The assay also incorporated two negative controls (for extraction and amplification), and two amplification-positive controls: a *Dolphin Morbillivirus* detected in a Risso’s dolphin (*Grampus griseus*) [39], for CeMV, and DNA from *B. abortus* (vaccine RB-51) [40], for *Brucella* spp. The obtained amplicons were purified using a commercial kit (Real Clean Spin kit 50 Test- REAL), following the manufacturer’s instructions, and subjected to Sanger DNA sequencing (Secugen S.L.,. Madrid, Spain). A BLAST search was conducted (www.ncbi.nlm.nih.gov/blast/Blast.cgi) to compare sequenced products with sequences described in GenBank for morbillivirus and *Brucella* spp.

**Fig. 2:**
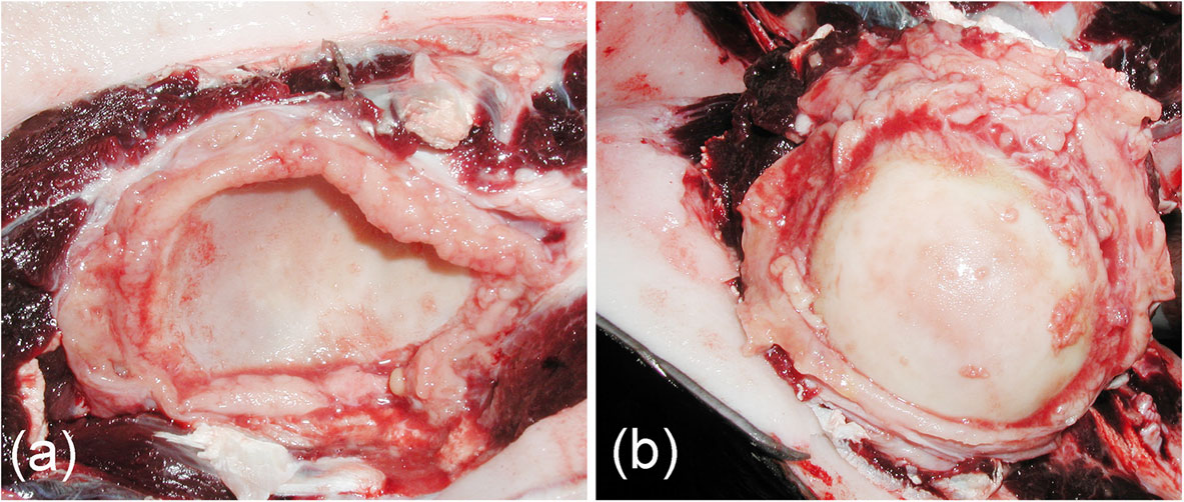
(Sero)fibrino-suppurative and proliferative arthritis of the shoulder joint. **a** view of the scapular glenoid fossa. **b** view of the humeral head

**Fig. 3:**
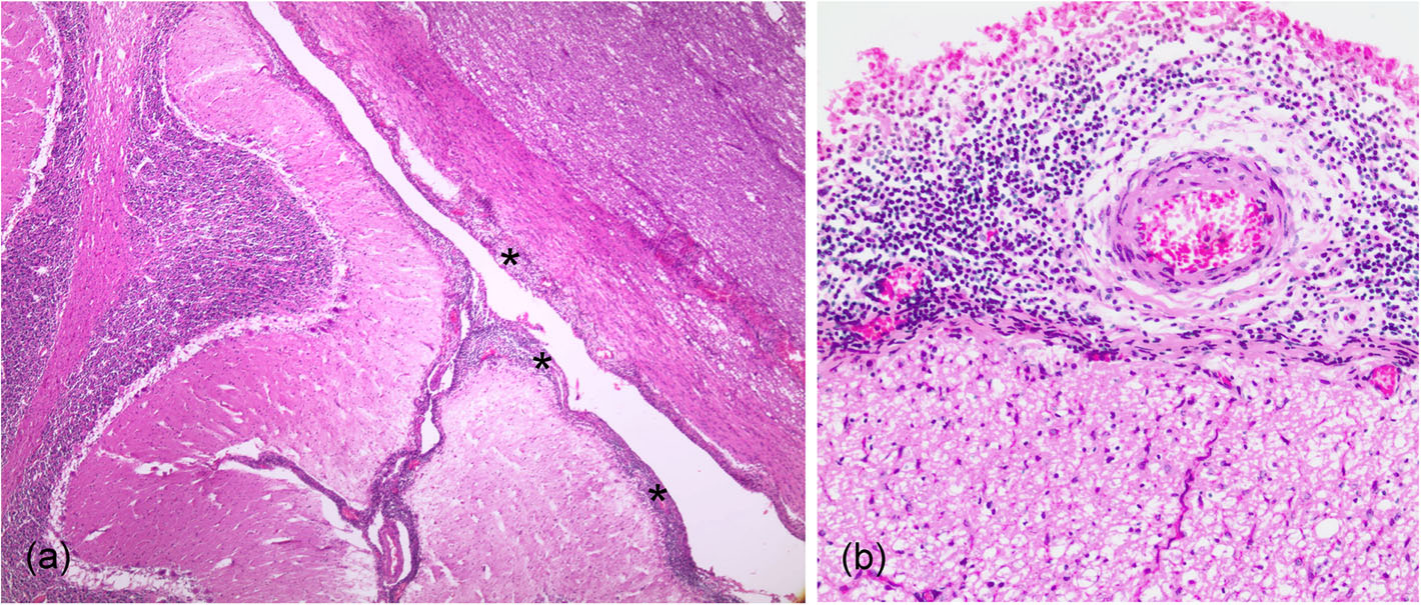
Meningoencephalitis. **a**. Mononuclear infiltrates in the meninges surrounding the cerebellum (asterisks). HE. 4x. **b**. Several layers of mononuclear cells in the meninges of the medulla oblongata. HE. 20x

Gross necropsy findings mainly comprised moderate-to-severe parasitic infestation in several anatomic locations, including verminous pneumonia by larvae and adult nematodes (morphologically identified as *Halocercus* spp. and *Stenurus* spp.). Additionally, thickened and congested cerebral leptomeninges and bilateral (sero)fibrino-suppurative and proliferative arthritis of the shoulder joints were detected (Fig. 2). Microscopic lesions were those typically observed in sub-acute systemic disease associated with CeMV infection [41], affecting lungs, lymph nodes, spleen, intestines, kidneys, pancreas, adrenal glands, and brain [34]. Immunohistochemistry and PCR showed evidence of the virus in all the affected tissues [34]. Histopathologically, lesions in the CNS mainly consisted of non-suppurative meningoencephalitis, ventriculitis and myelitis. More specifically, these findings consisted in marked, multifocal, subacute-to-chronic lymphohistiocytic meningoencephalitis with perivascular cuffing and gliosis in the cerebrum, cerebellum, spinal cord, and in the brainstem (pons). Additionally, the cerebrum and the brainstem (pons) presented granulomatous meningoencephalitis and ventriculitis with multinucleated giant cells and hemorrhage. The cerebrum also showed satellitosis, glial degeneration and necrosis, rarefaction/liquefactive necrosis, and vasculitis; the brainstem (pons) presented spongiosis and Wallerian degeneration; and the spinal cord showed evidence of glial nodules, white matter spongiosis and polyrradiculoneuritis. Finally, marked, multifocal, chronic lymphohistiocytic cranial neuritis, and meningitis and neurohypophisitis were present [42]. However – despite the severe non-suppurative meningitis (with more than 20 layers of histiocytic and lymphocytic cells), encephalomyelitis, and perineuritis in the CNS (Fig. 3) – immunopositivity against morbillivirus was only detected in some areas, with few lymphocytes, histiocytes, syncytial cells, and neurons, and some glial, and endothelial cells, showing positive immunostaining. Therefore, the nature of the inflammatory reaction, the lesion distribution pattern in the CNS, and the limited immunoreactivity, suggest a co-infection pathogen. The histopathological examination evidenced that the scapulohumeral synovium presented a severe, focally extensive, chronic necrosuppurative and granulomatous synovitis with fibroplasia, as well as rare bacteria and numerous multinucleated giant cells. These findings were consistent with previous descriptions of *Brucella* sp. infection in dolphins [1, 17, 19, 21, 22, 25, 26]. A 178-bp sequence length of the IS711 gene from the brain tissue was amplified (105-bp excluding primer length). This sequence showed a high similarity (99%) when comparing identical partial regions of larger fragments of IS711 with *Brucella* sp. detected in cetaceans (GenBank acc. no. KJ482569; CP006896-CP006899; AB126349; AF242532-AF242534). There was also a close similarity with *B. pinnipedialis* (GenBank acc. no. CP007742, CP007743, CP002078, and CP002079). However, *Brucella* strain ST27 delivered negative results. No amplification was obtained from the lung, spleen, and humeral joint samples. These preliminary results suggest that the subject of our study was infected by a marine *Brucella* strain. Nonetheless, further molecular characterisation will be necessary to better identify this strain.

**Fig. 4:**
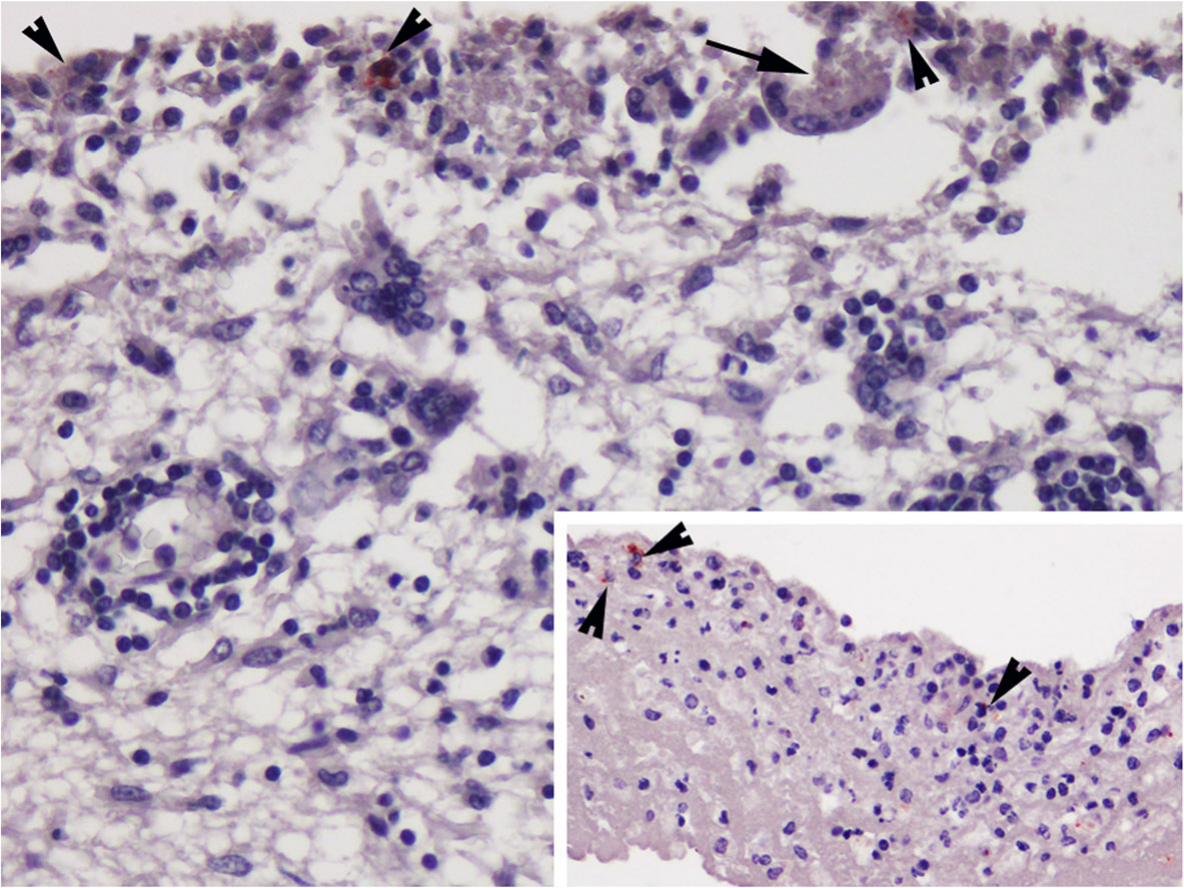
Immunohistochemical distribution of *Brucella* antigen in the central nervous system. Immunolabeling was mainly observed in the cytoplasm of isolated mononuclear phagocytic cells (arrowheads) and syncytia (arrows) in the meninges and adjacent areas of cerebral cortex. IHC, HE counterstaining. 60x. Inset: immunopositivity in some monocytic cells (arrowheads) inside meningeal vessels. IHC, HE counterstaining. 40x

*Brucella* sp. was detected by IHC in the CNS and in the shoulder joint. Immunostaining in the brain was observed in the cytoplasm of isolated mononuclear phagocytic cells in the meninges, inside meningeal vessels and in areas of cerebral cortex lying beneath the pia mater, which also presented a severe inflammation, mainly composed of macrophages and syncytia (with weak cytoplasmic immunoreaction) (Fig. 4). The antibody against morbillivirus showed the most intense immunopositivity in the same areas of the brain. Furthermore, immunostaining of the joint was located in the cytoplasm of isolated macrophages and multinucleated cells.

## Discussion and conclusions

**Fig. 5:**
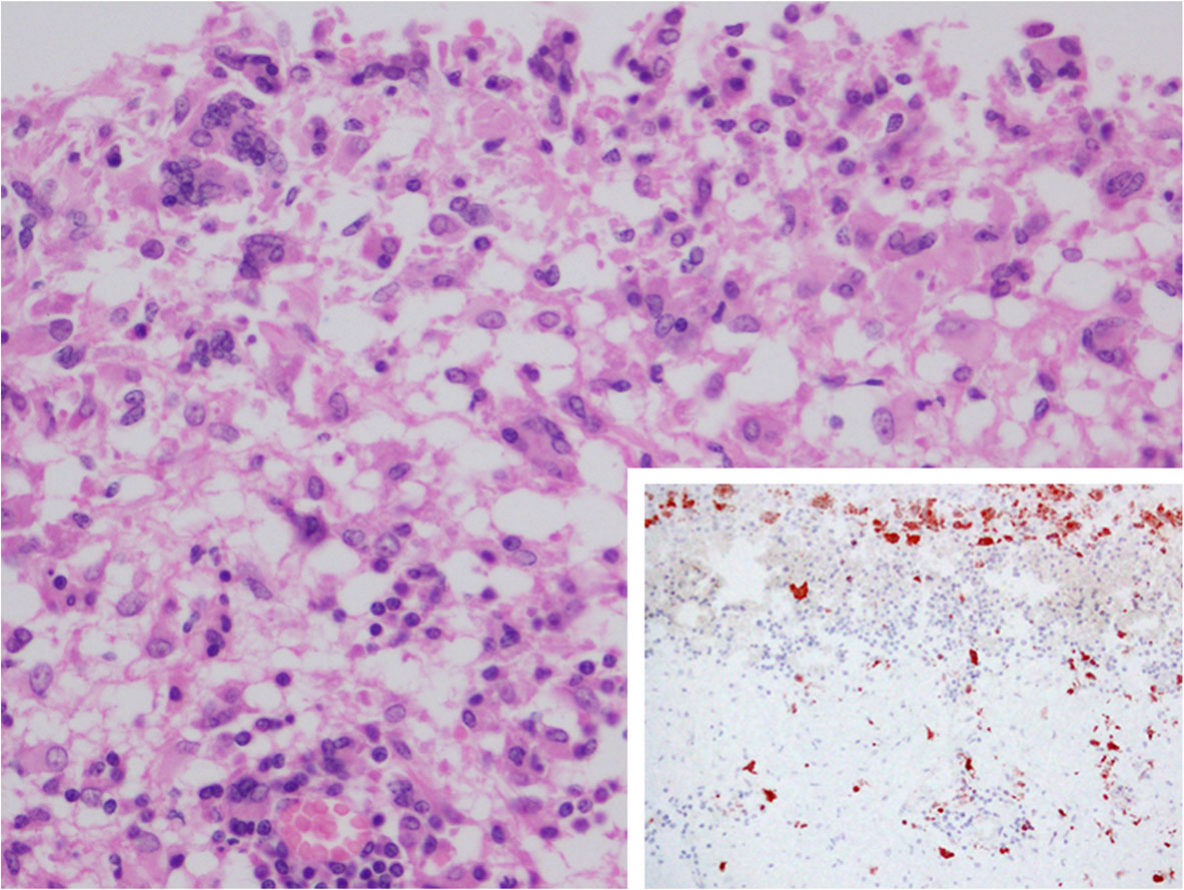
Central nervous system, cerebral cortex. Lymphohistiocytic and granulomatous meningoencephalitis with multinucleated giant cells, perivascular cuffing, gliosis and hemorrhages. 60x. Inset: Immunolabeling against morbillivirus was predominantly within the cytoplasm of macrophages and multinucleated giant cells (syncytia) in the same areas. IHC, HE counterstaining. 20x

Involvement of the CNS in *Brucella* spp.-infected cetaceans has been reported more frequently in striped dolphins, suggesting that this species is much more vulnerable to neurobrucellosis than others [1, 20]. Evidence of active *Brucella* spp. infection in the brain has been less frequently reported in other cetacean species [25–29]. PCR-confirmed *Brucella* meningoencephalitis has also been described in the common bottlenose dolphin species [30, 31], with some cases of CeMV co-infections [43]. *Cetacean Morbillivirus* is the most pathogenic virus threatening dolphins and whales worldwide, and common bottlenose and striped dolphins are among the species that are the most susceptible to CeMV infection [36, 44–52]. Depending on the stage of the infection, four presentation forms of the disease have been recently proposed: acute-, subacute-systemic diseases, chronic systemic infection and chronic localised CeMV encephalitis. During the first two stages of the disease, typical lesions are present, although they can be largely obscured by those produced by secondary pathogens as a consequence of profound immunosuppression. Secondary opportunistic pathogens usually include *Toxoplasma gondii*, herpesviruses, bacteria (such as *Photobacterium damselae*) and fungi (such as *Aspergillus fumigatus*) [41]. Interestingly, few cases of *Brucella* and CeMV co-infection have been reported previously [29, 40, 43, 53]. In those cases in which they were reported, more severe and extensive CNS inflammatory lesions were ascribed to *Brucella* sp. rather than to CeMV infection. Brain lesions related to *Brucella* sp. infection (neurobrucellosis) are typically located in the meninges and ependyma [1], while typical CeMV-associated brain lesions tend to involve the neuroparenchyma [41]. In our case, systemic lesions were indicative of a sub-acute CeMV infection, while the chronicity and inflammatory features of brain lesions were more suggestive of *Brucella* sp. infection. The DNA *Brucella* amplicon amplified around cycle 30, which also correlates with the weak positivity by IHC in the brain (low bacterial load). Thus, since the inflammatory response pattern was largely associated with *Brucella* infection (neurobrucellosis), in our opinion, *Brucella sp.* was the main cause of meningoencephalitis in this dolphin. The low immunolabeling against *Brucella* could be explained by the chronicity of the infection (bacterial clearance by the inflammatory reaction) [19] or the relative insensitivity of *Brucella* IHC in areas with few bacteria [54], as previously suggested. However, CeMV also likely played a role, although those typical lesions were overlapped, masked, and/or non-evident. In contrast to previous reports, positive immunolabeling for morbillivirus and for *Brucella* by IHC were present in the CNS of the bottlenose dolphin from our study. Both positive immunolabeling also appeared in those areas where more macrophages (recognized cell target for *Brucella* and morbillivirus) and syncytia were present (Fig. 5). The latter finding could be interpreted as a reactivation of the *Brucella* infection in the brain (potentiated by the immune suppressive properties of CeMV), which could move morbillivirus-infected macrophages to the site, spreading the virus into the CNS. However, how or whether an interaction exists between these two CNS pathogens is unknown. Another question that arose was how this animal got infected. It has been suggested that *Brucella* spp. infection can be transmitted horizontally (by sexual intercourse, maternal feeding, placental tissues or aborted fetuses) or vertically (from mother to the fetus). Other proposed transmission routes are through fish or helminth reservoirs [20, 21, 55, 56]. The estimated age of the subject and the course of the infection could evidence an in utero infection. This case adds a new record of *Brucella* sp. infection in the common bottlenose dolphin, a species in which reported cases of brucellosis are particularly scarce, despite the serological and molecular evidence of *Brucella* exposure reported both in free-ranging and under human care specimens worldwide [6, 9, 10, 13, 38, 57–59]. This is the first detection of *Brucella* sp. in a cetacean stranded in the Canary Islands, a region with no reported cases of brucellosis for this taxon. The only evidence of brucellosis in the archipelago is a low prevalence of antibodies against *Brucella* sp. (1%) described in camels [60, 61]. The zoonotic potential of *B. ceti*, particularly the strain ST27 [62–64], has been previously discussed. Specifically, 4 human cases of acquired infection (3 natural and 1 laboratorial) by *Brucella* sp. from marine mammals, sharing the same genotype (ST27), based on multilocus sequence typing, have been reported [64–67]. The presence of this strain has been detected in cetaceans from the Pacific waters [1, 4, 38] and more recently in a common bottlenose dolphin from the northern Adriatic Sea found in Croatia [59]. Thus, special precautions should be taken when these marine mammal species are being handled (rescue, rehabilitation, necropsy and lab procedures).

## Data Availability

The datasets used and/or analysed during the current study are available from the corresponding author on reasonable request.

## Abbreviations

bp: Base pairs
CDV: Canine distemper virus
CeMV: *Cetacean Morbillivirus*
FFPE: Formalin-fixed, paraffin-embedded
HE: Hematoxylin and eosin
IHC: Immunohistochemistry
P gene: Phosphoprotein gene
PCR: Polymerase chain reaction
